# Expanding the clinical spectrum of Lemierre syndrome and Lemierre-like septic internal jugular vein thrombophlebitis: a four-case series

**DOI:** 10.3389/fmed.2026.1821698

**Published:** 2026-09-23

**Authors:** Abdullah Alyousef, Yacoub Abuzied, Abdullah Alhassoun, Lama Alfehaid, Mohammed AlSheef

**Affiliations:** 1 Internal Medicine and Thrombosis, Medical Specialties Department, King Fahad Medical City, Riyadh Second Health Cluster, Riyadh, Saudi Arabia; 2 Department of Nursing, Rehabilitation Hospital, King Fahad Medical City, Riyadh Second Health Cluster, Riyadh, Saudi Arabia; 3 Department of Nursing, Faculty of Medicine and Health Sciences, Universiti Putra Malaysia, Serdang, Malaysia; 4 Department of Pharmacy Practice, College of Pharmacy, King Saud bin Abdulaziz University for Health Sciences, Riyadh, Saudi Arabia; 5 Pharmaceutical Care Department, King Abdulaziz Medical City, Riyadh, Saudi Arabia; 6 King Abdullah International Medical Research Center, Riyadh, Saudi Arabia; 7 College of Medicine, Alfaisal University, Riyadh, Saudi Arabia

**Keywords:** anticoagulation, bacteremia, internal jugular vein thrombosis, Lemierre syndrome, septic thrombophlebitis

## Abstract

**Introduction:**

Lemierre syndrome is a rare but potentially life-threatening condition, characterized by septic thrombophlebitis of the internal jugular vein and classically following an oropharyngeal infection. Although traditionally associated with young, otherwise healthy adults and *Fusobacterium necrophorum* infection, contemporary reports increasingly recognize a broader clinical spectrum, including culture-negative and Lemierre-like presentations.

**Case presentation:**

We describe four patients with classic Lemierre syndrome or Lemierre-like septic internal jugular vein thrombophlebitis managed between 2012 and 2023 at a tertiary care center in Saudi Arabia. Patients ranged in age from 31 to 72 years and had diverse underlying comorbidities. All presented with unilateral neck pain or swelling, and imaging confirmed internal jugular vein thrombosis in all four cases, including one with intracranial extension into the sigmoid and transverse venous sinuses. Deep neck abscesses were identified in three patients. Blood cultures were negative in all cases, while an abscess culture from one patient with poorly controlled diabetes mellitus yielded *Klebsiella pneumoniae*. Antimicrobial therapy ranged from 3 to 6 weeks. Anticoagulation strategies varied according to thrombus extent, bleeding risk, comorbidities, and overall clinical presentation. All patients experienced favorable clinical outcomes, with follow-up imaging demonstrating thrombus stability, partial recanalization, or complete resolution.

**Conclusion:**

This case series illustrates the broader clinical spectrum of septic internal jugular vein thrombophlebitis, encompassing both classic Lemierre syndrome and Lemierre-like presentations. The cases demonstrate that diagnosis may rely predominantly on compatible clinical and radiological findings despite negative blood cultures. Management should be individualized according to disease severity, thrombus extent, bleeding risk, and patient-specific factors, particularly given the limited evidence guiding anticoagulation in this setting.

## Introduction

Lemierre syndrome is a rare infectious disease first described by Dr. André Lemierre in 1936, after whom it is named (1). It is also referred to as necrobacillosis or postanginal sepsis and predominantly affects young, otherwise healthy adults. In a large review of more than 220 patients, most were male with a median age of 20 years (2).

The syndrome is most commonly caused by *Fusobacterium necrophorum*, an anaerobic gram-negative bacterium that forms part of the normal oropharyngeal flora (3). However, other pathogens, such as *Staphylococcus aureus* and *Klebsiella pneumoniae*, have also been reported (4). Lemierre syndrome typically follows an episode of acute pharyngitis or tonsillitis and initially presents with fever, sore throat, odynophagia, dysphagia, neck pain, and signs of systemic infection. Septic thrombophlebitis of the internal jugular vein (IJV) usually develops within 1–3 weeks after the primary infection. Pulmonary septic emboli represent the most common metastatic complication, followed by involvement of large joints such as the knees and hips (2–6).

Because of its rarity and variable clinical presentation, diagnosis requires a high index of suspicion, particularly in patients presenting with persistent fever and unilateral neck pain following a recent oropharyngeal infection. Imaging studies and microbiological evaluation are essential for confirming the diagnosis. Blood cultures may be negative, particularly in patients who have received prior antibiotics or when anaerobic organisms are difficult to isolate (4).

Prolonged antibiotic therapy with adequate anaerobic coverage remains the cornerstone of treatment. Metronidazole is frequently used and is typically administered for up to 6 weeks, although the optimal duration of therapy remains uncertain (4). Most patients receive intravenous antibiotics during hospitalization, followed by oral therapy to complete a total treatment course of approximately 6 weeks (7).

The role of anticoagulation in Lemierre syndrome remains controversial due to the absence of randomized controlled trials. Anticoagulation is generally considered in selected cases, including persistent fever, thrombus progression, or clinical deterioration (2). However, a 2020 meta-analysis found no significant difference in vessel recanalization between anticoagulated and non-anticoagulated patients (8). Consequently, treatment decisions are often individualized. Advances in antimicrobial therapy notwithstanding, Lemierre syndrome remains associated with significant morbidity, with reported mortality rates ranging from 10% to 15% (9).

Given the rarity of Lemierre syndrome and the expanding spectrum of its clinical presentations, additional case reports remain valuable for improving disease recognition and informing management strategies. Although classic Lemierre syndrome is traditionally characterized by antecedent oropharyngeal infection, *Fusobacterium bacteremia*, internal jugular vein thrombosis, and metastatic septic emboli, contemporary reports increasingly describe incomplete or Lemierre-like presentations lacking one or more of these defining features. Such atypical presentations pose diagnostic challenges and contribute to ongoing uncertainty about optimal management, particularly regarding antimicrobial duration and the role of anticoagulation. In this study, we present four patients representing the spectrum of classic Lemierre syndrome and Lemierre-like septic internal jugular vein thrombophlebitis managed at a tertiary care center in Saudi Arabia. These cases highlight the diagnostic challenges of culture-negative disease, the occurrence of septic internal jugular vein thrombophlebitis outside the classic demographic profile, and the need for individualized therapeutic strategies based on clinical presentation and disease extent.

### Timeline of clinical events

The overall clinical course of the four cases is summarized in Table 1, which illustrates the sequence of infection, diagnosis, treatment initiation, and follow-up outcomes.

**Table 1:** Timeline of clinical events according to CARE guidelines.

Case	Preceding event	Presenting symptoms	Diagnostic imaging	Microbiological findings	Treatment initiated	Follow-up outcome
Case 1	Permcath-associated deep neck infection	Fever, right-sided neck swelling, and localized neck pain	CT neck showing retropharyngeal abscess and right IJV thrombosis	Blood cultures negative	IV piperacillin/tazobactam for 2 weeks, followed by oral amoxicillin/clavulanate; apixaban 2.5mg twice daily, planned for 3 months	Resolution of retropharyngeal infection at 2 weeks with persistent IJV thrombosis; complete thrombus resolution on subsequent follow-up imaging
Case 2	Progressive deep neck infection	Left-sided neck swelling, pain, and dysphagia	CT neck showing multiloculated abscess and left IJV occlusion	Blood cultures negative	IV piperacillin/tazobactam followed by oral amoxicillin/clavulanate; prophylactic-dose enoxaparin	Partial recanalization of the left IJV on follow-up ultrasound
Case 3	Upper respiratory tract infection approximately 1 month earlier	Left-sided neck pain and swelling	CT and ultrasound confirming left IJV thrombosis	Blood cultures negative	Oral amoxicillin/clavulanate and apixaban 5 mg for 3 months	Stable IJV thrombus without progression
Case 4	Oropharyngeal infection	Fever, right-sided neck swelling, dysphagia, and voice changes	CT neck showing carotid space abscess and right IJV thrombosis with intracranial extension	Blood cultures negative; abscess culture positive for *Klebsiella pneumoniae*	Surgical drainage, antibiotics for 6 weeks, and warfarin for 6 weeks	Complete thrombus resolution at 6-week follow-up

CT, computed tomography; IJV, internal jugular vein; IV, intravenous; MRI, magnetic resonance imaging; US, ultrasonography; DM, diabetes mellitus.

## Case descriptions

Cases were identified through review of the thrombosis clinic registry and electronic medical records at King Fahad Medical City between 2012 and 2023. Patients were included if they had radiologically confirmed internal jugular vein thrombosis associated with deep neck infection or septic thrombophlebitis, with clinical features compatible with Lemierre syndrome or Lemierre-like syndrome.

### Case 1

A 51-year-old man with a history of ischemic heart disease, end-stage renal disease, and mantle cell lymphoma presented to the Emergency Department with a 2-day history of right-sided neck swelling, fever, and localized pain at the site of a tunneled dialysis catheter (Permcath) inserted approximately 1 month earlier.

Blood cultures obtained on presentation were negative. Laboratory findings at admission are summarized in Table 2.

**Table 2:** Laboratory profile of Case 1 at admission.

Test	Value at initial presentation
WBC	14.03 × 10^9^/L
Hb	11.9 g/dL
PLT	281 × 10^9^/L
Scr	1,142 μmol/L
Na	131 mmol/L
K	4.35 mmol/L
ESR	120 mm/h
CRP	171 mg/L
HbA1c	4.9%

WBC, white blood cell count; Hb, hemoglobin; PLT, platelet count; SCr, serum creatinine; Na, sodium; K, potassium; ESR, erythrocyte sedimentation rate; CRP, C-reactive protein; HbA1c, glycated hemoglobin.

Contrast-enhanced computed tomography (CT) of the neck demonstrated retropharyngeal soft-tissue infection with phlegmon formation and an abscess extending from C1 to C5–C6, causing mild airway narrowing without obstruction. CT also revealed enlargement and thrombosis of the right internal jugular vein, accompanied by marked perivascular inflammatory changes, consistent with septic thrombophlebitis (Figure 1).

**Figure 1:**
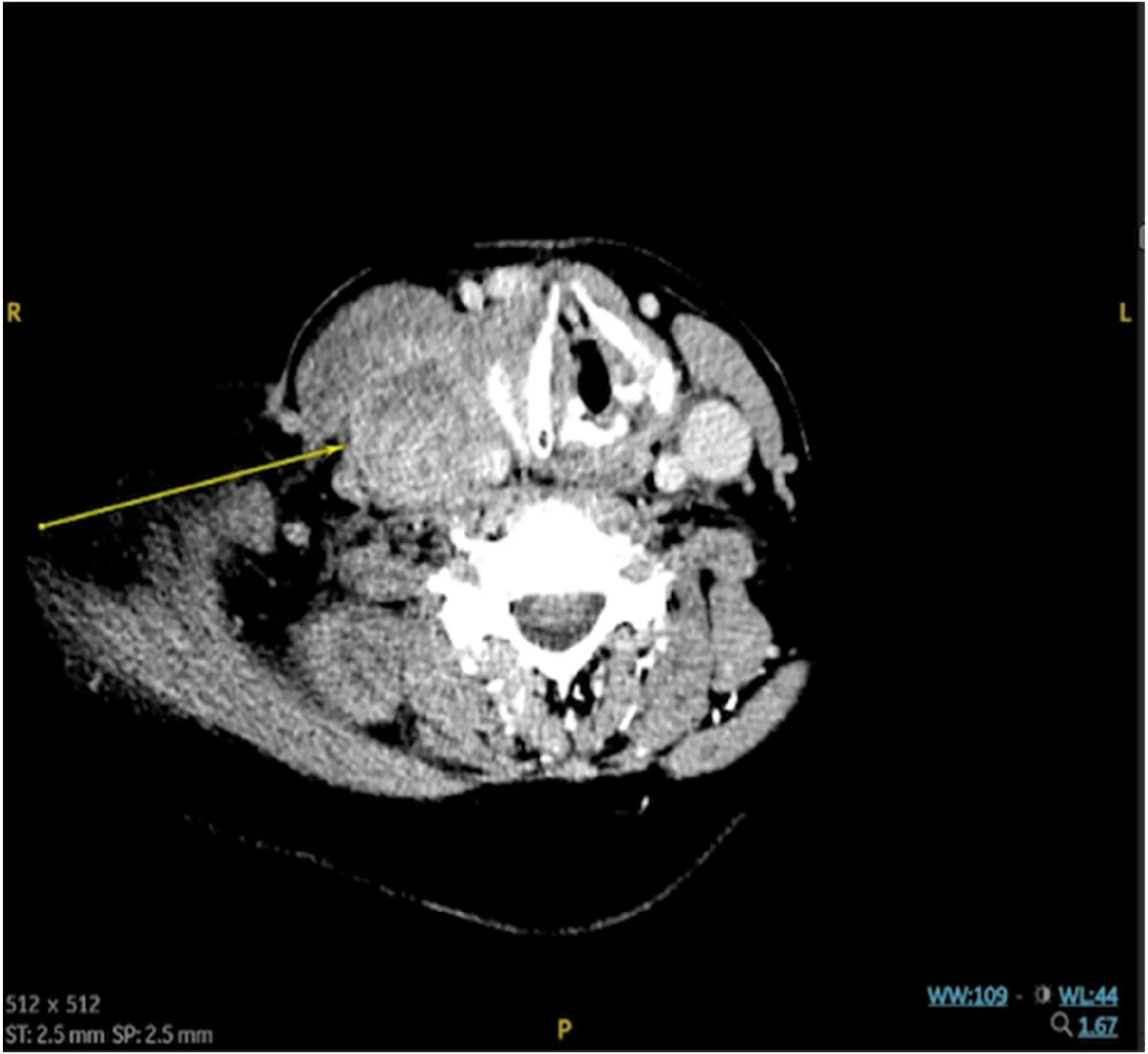
Contrast-enhanced CT of the neck demonstrating retropharyngeal soft-tissue swelling and fluid collection consistent with soft-tissue infection/abscess formation. There is enlargement of the right internal jugular vein extending to the right brachiocephalic vein with a large intraluminal thrombus measuring more than 8 cm in length.

Image-guided aspiration of the retropharyngeal collection was attempted unsuccessfully by the Interventional Radiology team. The patient was treated with intravenous piperacillin/tazobactam for 2 weeks, followed by oral amoxicillin/clavulanate for an additional 2 weeks, completing a total antimicrobial treatment duration of 4 weeks. In view of the septic right internal jugular vein thrombosis, anticoagulation with apixaban 2.5 mg twice daily was initiated, with a planned treatment duration of 3 months.

At the 2-week follow-up, contrast-enhanced CT demonstrated interval resolution of the retropharyngeal soft-tissue infection and abscess; however, residual thrombosis of the right internal jugular vein persisted (Figure 2). Follow-up imaging approximately 9 months after the initial presentation demonstrated complete resolution of the thrombosis.

**Figure 2:**
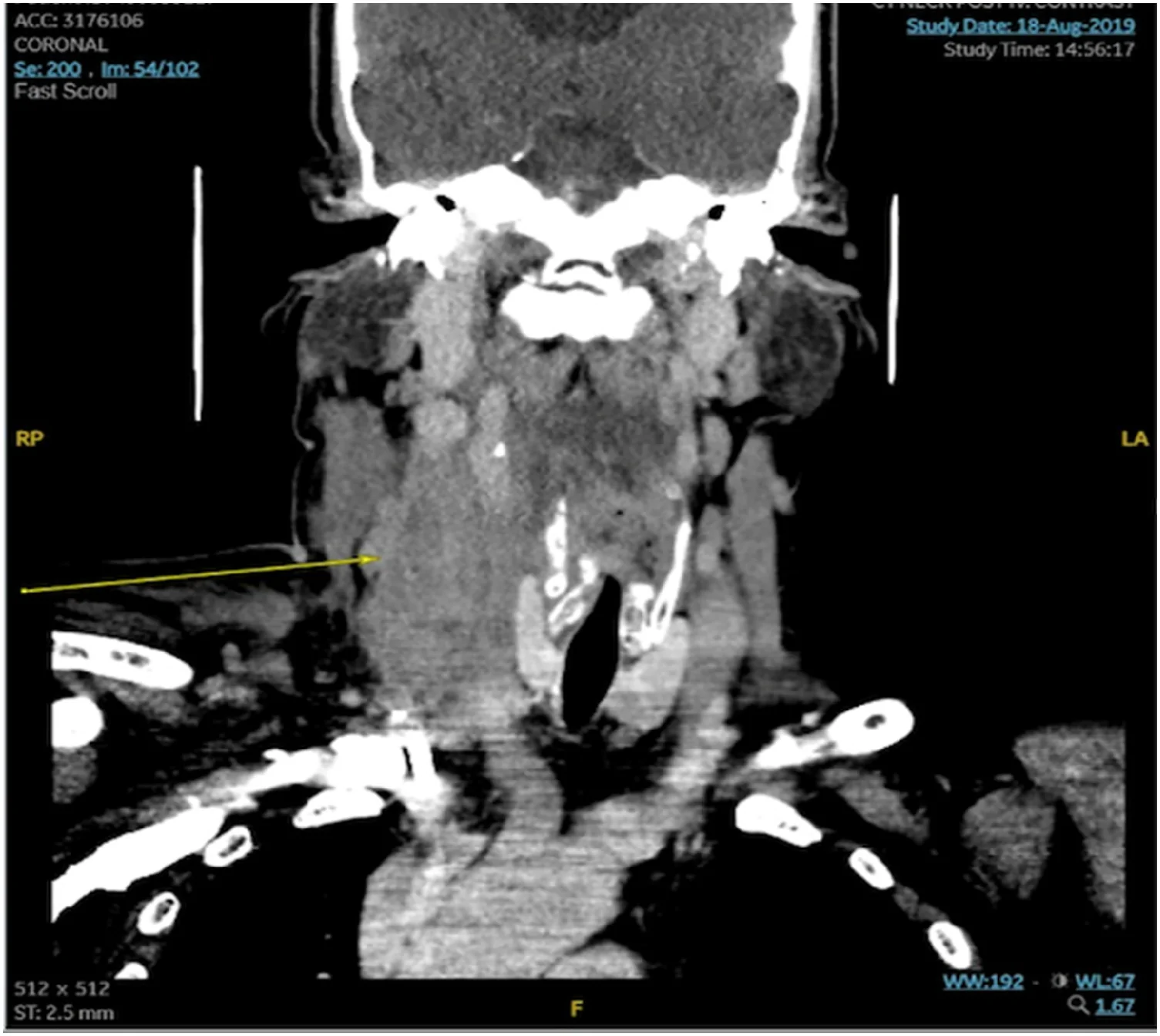
Follow-up contrast-enhanced CT of the head and neck obtained 2 weeks after initiation of treatment. Interval resolution of the retropharyngeal soft-tissue infection/abscess is noted. Persistent thrombosis of the right internal jugular vein remains evident.

Given the close temporal relationship between Permcath insertion and symptom onset, and the absence of a preceding oropharyngeal infection, the clinical presentation was considered most consistent with catheter-associated septic internal jugular vein thrombophlebitis, representing a Lemierre-like syndrome.

### Case 2

A 31-year-old woman with a history of megaloblastic anemia, prior cerebral aneurysm, and bilateral lung nodules presented to the Emergency Department with progressive left anterior neck swelling of 1 week's duration. The swelling was painful, tender, firm, and associated with intermittent erythema. She also reported dysphagia but denied purulent discharge. Vital signs were stable at presentation. Laboratory findings at admission are summarized in Table 3.

**Table 3:** Laboratory profile of Case 2 at admission.

Test	Value at initial presentation
WBC	7 × 10^9^/L
Hb	10.2 g/dL
PLT	259 × 10^9^/L
Scr	42 μmol/L
Na	132 mmol/L
K	3.17 mmol/L
ESR	116 mm/h
CRP	98.5 mg/L
HbA1c	5.2%

WBC, white blood cell count; Hb, hemoglobin; PLT, platelet count; SCr, serum creatinine; Na, sodium; K, potassium; ESR, erythrocyte sedimentation rate; CRP, C-reactive protein; HbA1c, glycated hemoglobin.

Blood cultures were obtained and were negative. Contrast-enhanced CT of the neck demonstrated a multiloculated abscess within the left anterior neck involving the sternocleidomastoid muscle, with associated occlusion of the left internal jugular vein (Figures 3A,B).

**Figure 3:**
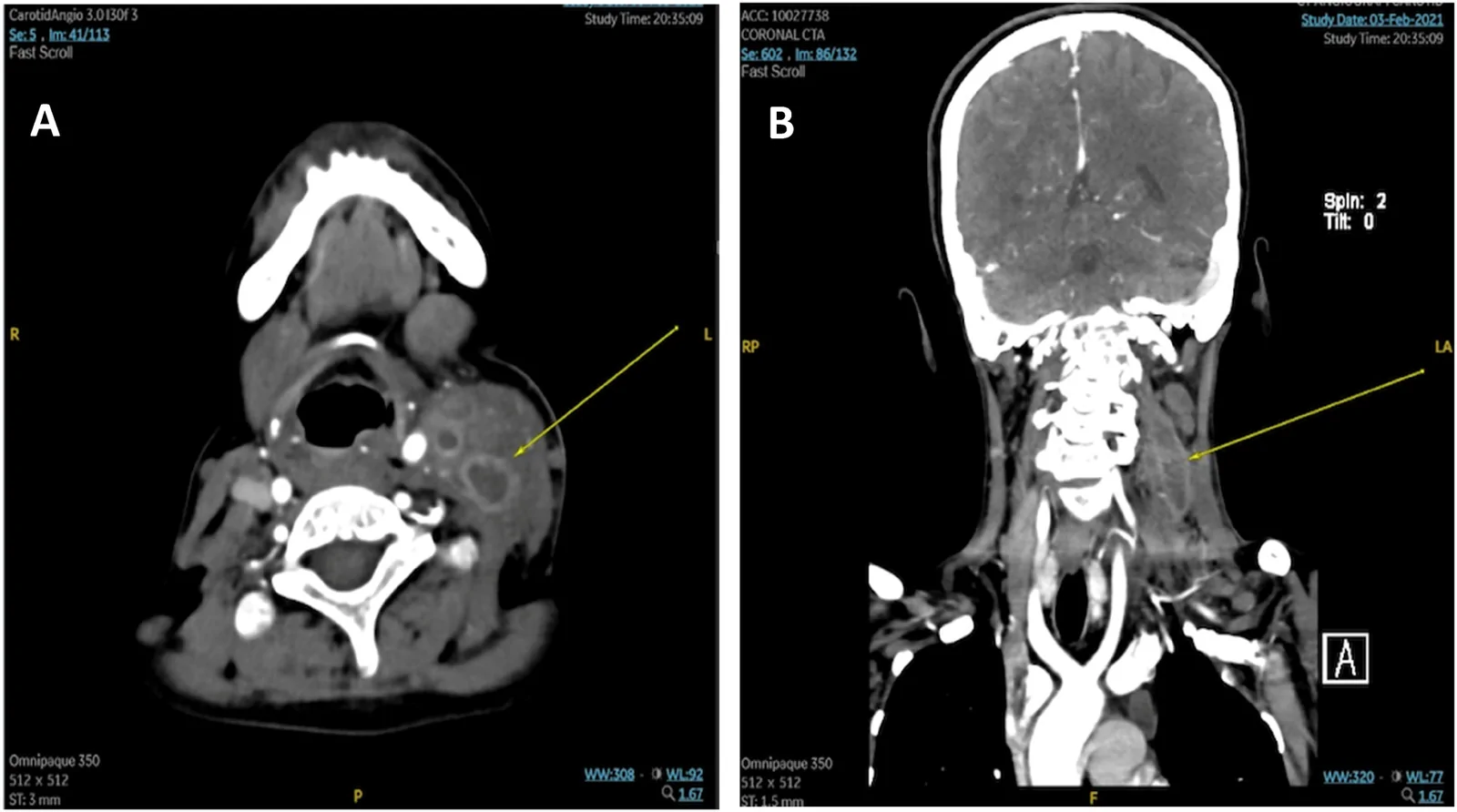
**(A,B)** Contrast-enhanced CT of the neck (axial and coronal views) demonstrating a multiloculated, peripherally rim-enhancing collection within the anterior left neck, located deep to the sternocleidomastoid muscle. The collection is occluding the left internal jugular vein just proximal to its drainage. There is associated marked subcutaneous edema, surrounding inflammatory changes, and fat stranding.

The abscess was located deep within the neck, making aspiration or surgical drainage technically challenging; therefore, no drainage procedure was performed.

The patient was started on intravenous piperacillin/tazobactam for 8 days, followed by oral amoxicillin/clavulanate for an additional 10 days (total duration approximately 3 weeks). She also received prophylactic-dose enoxaparin during hospitalization.

After 7 days of antibiotic therapy, a follow-up neck ultrasound demonstrated interval regression of the left-sided collection, with a residual collection measuring approximately 0.9 cm × 0.5 cm. Partial recanalization of the left internal jugular vein thrombosis was observed (Figure 4), consistent with clinical improvement.

**Figure 4:**
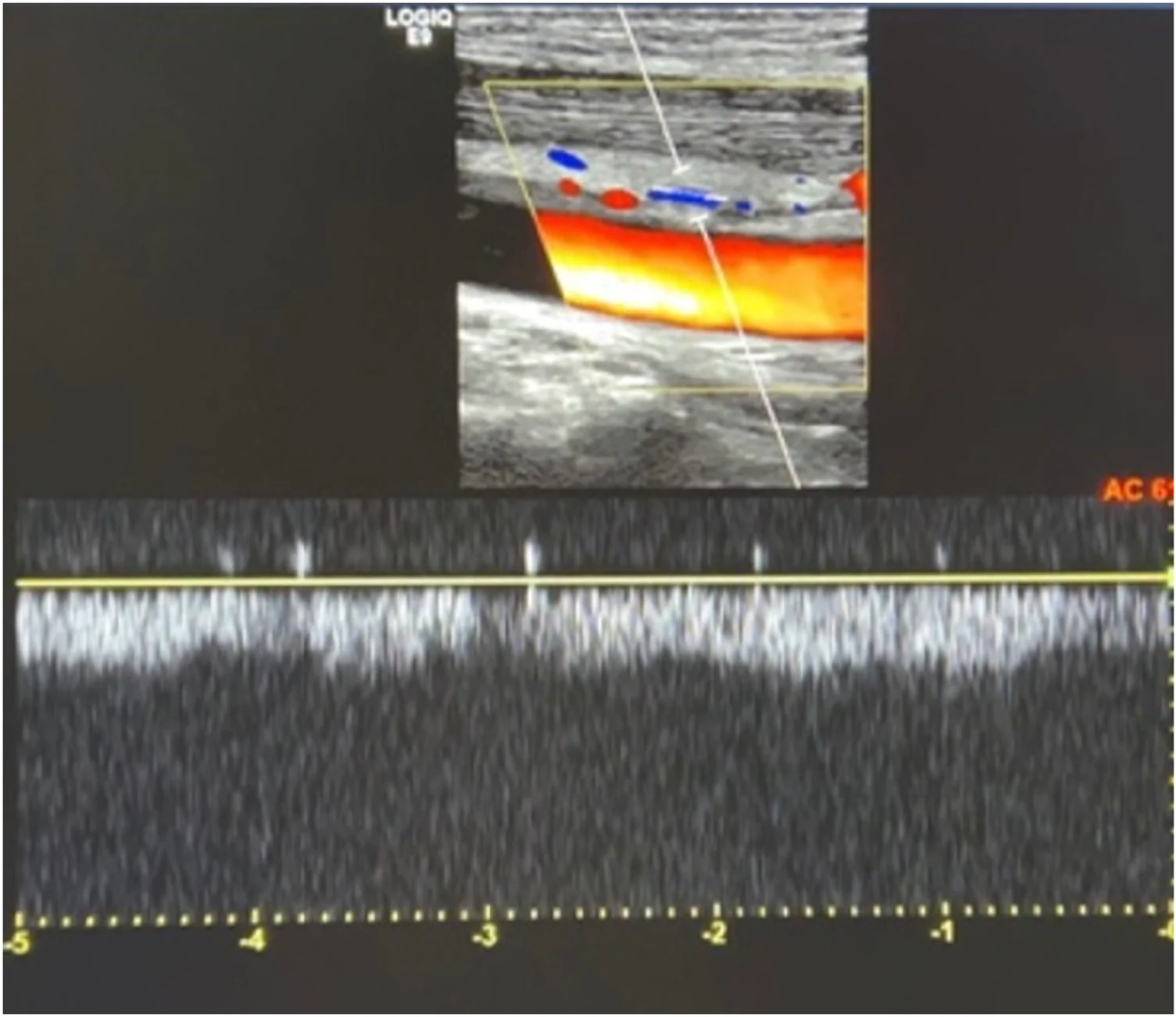
Follow-up ultrasound of the neck showing interval regression of the left sternocleidomastoid collection, with a residual collection measuring approximately 0.9 cm × 0.5 cm. Persistent thickening and edema of the sternocleidomastoid muscle are noted. Partial recanalization of the previously documented left internal jugular vein thrombosis is observed.

### Case 3

A 72-year-old woman with a history of diabetes mellitus, coronary artery disease, and atrial fibrillation presented to the Emergency Department with a 2-week history of left-sided neck pain and swelling. She reported that her symptoms developed approximately 1 month after an upper respiratory tract infection. Vital signs were stable at presentation.

Blood cultures were obtained and returned negative. Laboratory findings at admission are summarized in Table 4.

**Table 4:** Laboratory profile of Case 3 at admission.

Test	Value at initial presentation
WBC	3.73 × 10^9^/L
Hb	13.8 g/dL
PLT	256 × 10^9^/L
Scr	70 μmol/L
Na	133 mmol/L
K	3.9 mmol/L
ESR	99 mm/h
CRP	9.5 mg/L
HbA1c	10.56%

WBC, white blood cell count; Hb, hemoglobin; PLT, platelet count; Scr, serum creatinine; Na, sodium; K, potassium; ESR, erythrocyte sedimentation rate; CRP, C-reactive protein; HbA1c, glycated hemoglobin.

Contrast-enhanced CT of the neck demonstrated circumferential wall thickening of the left internal jugular vein with marked surrounding fat stranding and associated luminal narrowing, findings consistent with thrombophlebitis (Figures 5A,B).

**Figure 5:**
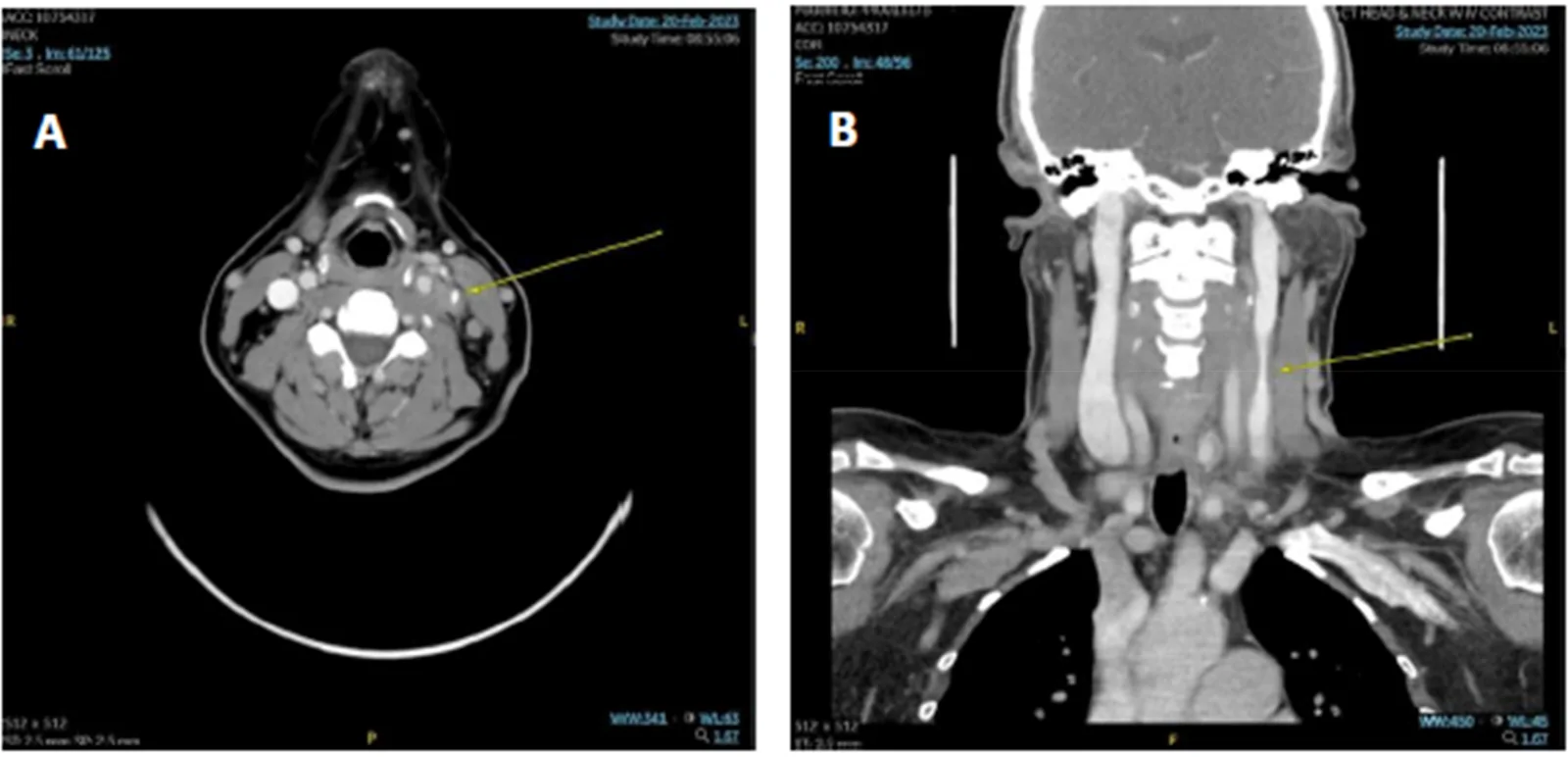
**(A,B)** Contrast-enhanced CT of the neck demonstrating diffuse circumferential wall thickening of the left internal jugular vein with marked surrounding fat stranding. Multiple areas of luminal narrowing are present, consistent with thrombophlebitis.

Ultrasound examination further confirmed circumferential thrombosis of the left internal jugular vein with a partially patent central lumen and reduced compressibility (Figure 6), consistent with partial thrombosis.

**Figure 6:**
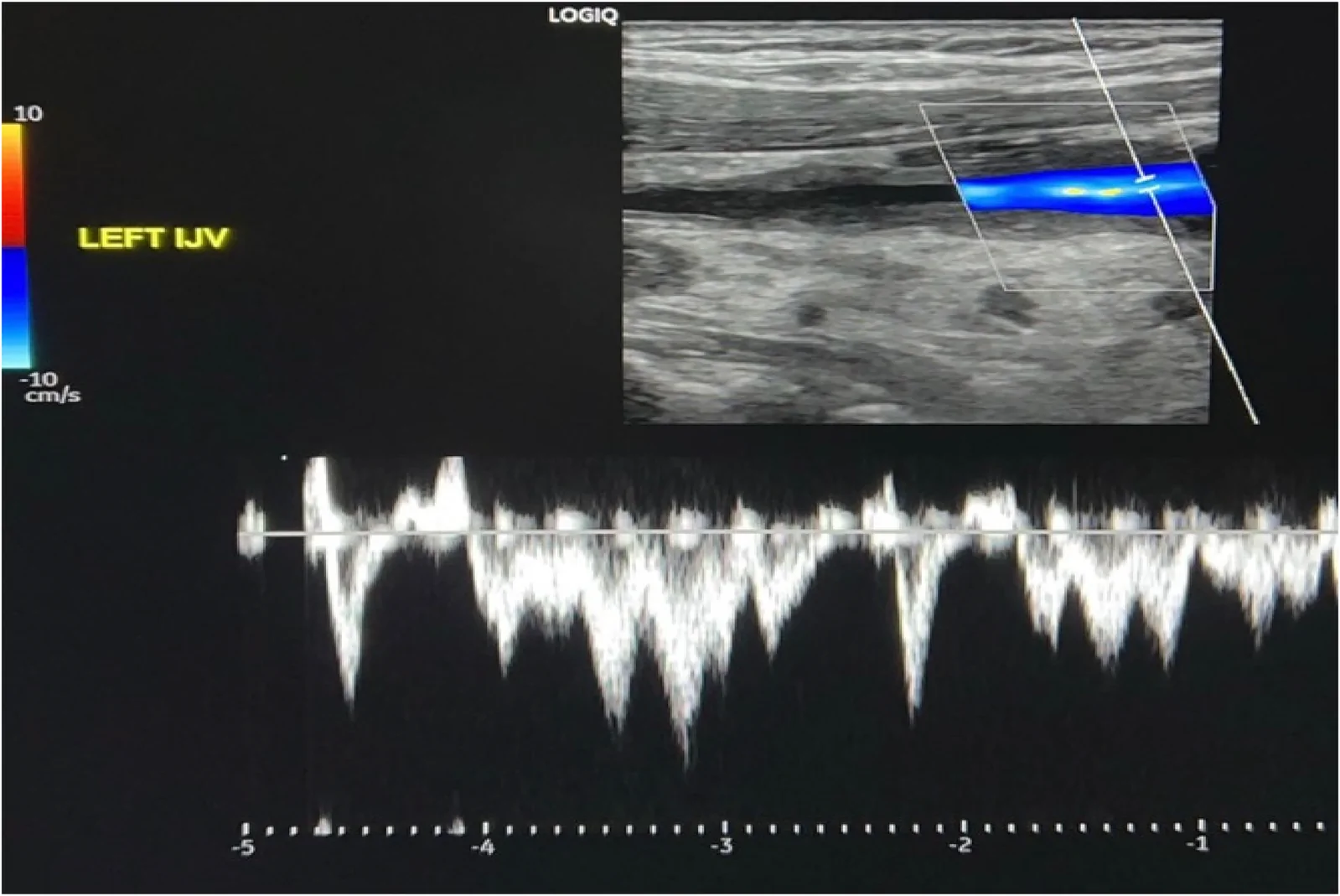
Ultrasound of the neck demonstrating circumferential thrombosis of the left internal jugular vein with a partially patent central lumen and reduced compressibility, consistent with partial thrombosis. The right internal jugular vein appears patent and fully compressible.

The patient was treated with oral amoxicillin/clavulanate for 3 weeks. Anticoagulation with apixaban 5 mg orally twice daily was initiated and continued for a total duration of 3 months. Clinical stability was maintained, and follow-up imaging demonstrated a stable thrombus without evidence of progression. No bleeding complications were observed during anticoagulation therapy.

### Case 4

A 63-year-old man with a 47-year history of poorly controlled diabetes mellitus presented to the Emergency Department with a 5-day history of right-sided neck swelling and fever. The swelling progressively increased in size and was associated with pain, dysphagia, odynophagia, voice changes, and decreased appetite. Laboratory findings at admission are summarized in Table 5.

**Table 5:** Laboratory profile of Case 4 at admission.

Test	Value at initial presentation
WBC	10.5 × 10^9^/L
Hb	13.9 g/dL
PLT	479 × 10^9^/L
Scr	90 μmol/L
Na	133 mmol/L
K	3.9 mmol/L
ESR	44 mm/h
CRP	9.5 mg/L
HbA1c	9.9%

WBC, white blood cell count; Hb, hemoglobin; PLT, platelet count; Scr, serum creatinine; Na, sodium; K, potassium; ESR, erythrocyte sedimentation rate; CRP, C-reactive protein; HbA1c, glycated hemoglobin.

Blood cultures were obtained and remained negative. The patient underwent incision and drainage of the neck abscess without complication. Culture and sensitivity testing of the abscess content grew *K. pneumoniae*.

Contrast-enhanced CT of the neck demonstrated a multiloculated abscess with irregular peripheral enhancement within the right carotid space. The palatine tonsils were enlarged, more pronounced on the right, with small areas of low attenuation. Imaging also revealed thrombosis of the right internal jugular vein with associated wall enhancement (Figure 7). The thrombus extended intracranially into the right sigmoid and transverse venous sinuses and inferiorly to the level of C7.

**Figure 7:**
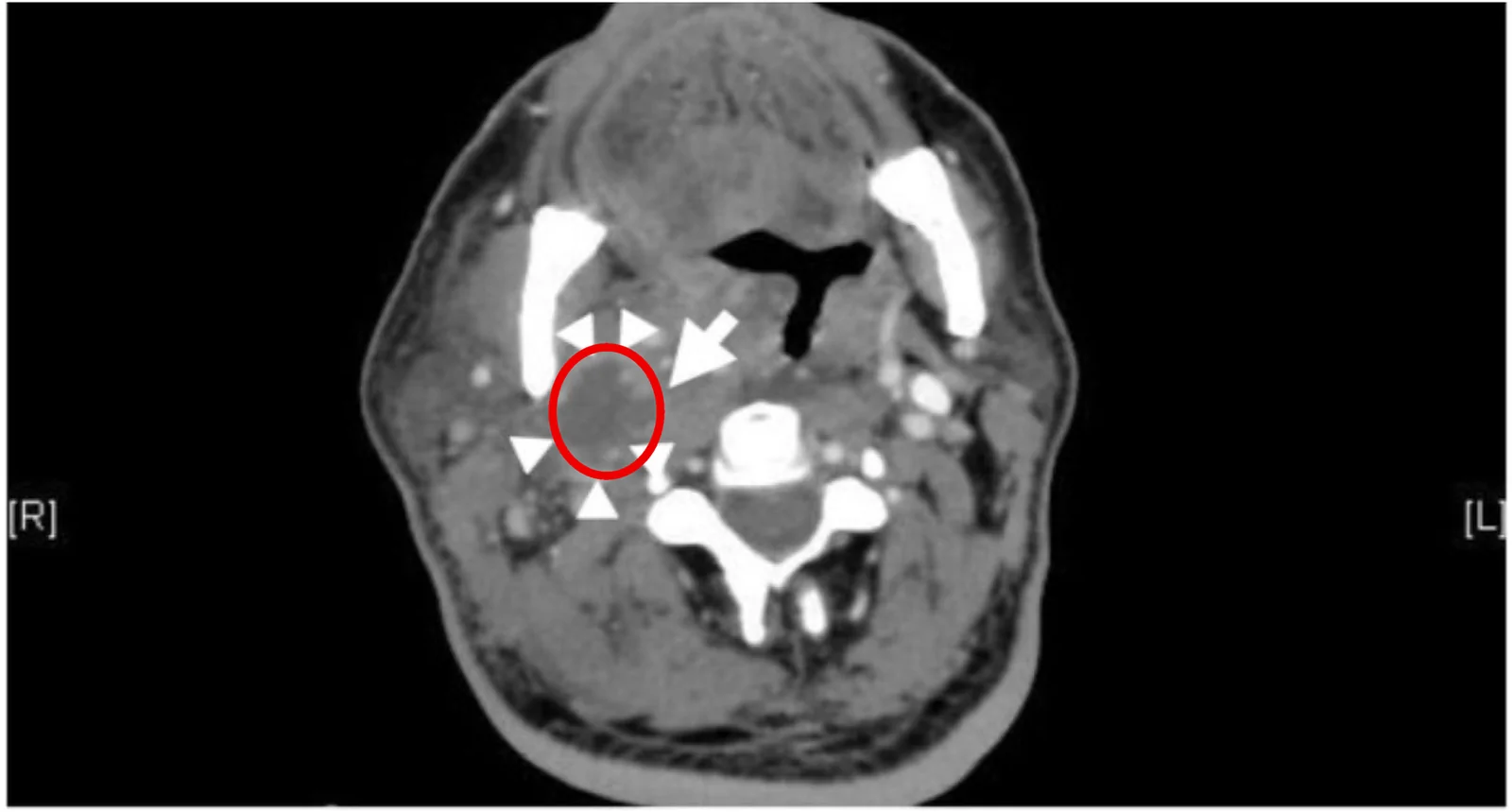
Contrast-enhanced CT of the neck demonstrating a multiloculated abscess within the right carotid space (circled). Thrombosis of the right internal jugular vein (arrow) is present, with thrombus extension noted.

The patient was treated with clindamycin and cefuroxime for a total duration of 6 weeks. Anticoagulation with warfarin was initiated concurrently and continued for 6 weeks to manage the internal jugular vein thrombosis. Follow-up neck ultrasonography performed 6 weeks later demonstrated complete resolution of the thrombosis.

### Summary of cases

The overall clinical characteristics, microbiological findings, and management strategies for all four patients are summarized in Table 6, highlighting the variability in patient demographics, clinical presentation, and therapeutic approaches.

**Table 6:** Summary of clinical characteristics and management of the four cases.

Variable	Case 1	Case 2	Case 3	Case 4
Classification	Lemierre-like syndrome (catheter-associated septic IJV thrombophlebitis)	Lemierre-like syndrome	Lemierre-like syndrome	Classic Lemierre syndrome
Gender	Male	Female	Female	Male
Age	51 years	31 years	72 years	63 years
Comorbidities	Ischemic heart disease, ESRD, mantle cell lymphoma	Megaloblastic anemia, prior cerebral aneurysm	Diabetes mellitus, CAD, atrial fibrillation	Long-standing poorly controlled diabetes mellitus
Preceding event	Permcath insertion with catheter-associated deep neck infection	Deep neck infection	Upper respiratory tract infection approximately 1 month earlier	Oropharyngeal infection
Presentation	Right-sided neck swelling, fever, and localized neck pain	Left-sided neck swelling, pain, erythema, and dysphagia	Left-sided neck pain and swelling	Right-sided neck swelling, fever, dysphagia, and voice changes
Diagnostic imaging	Contrast-enhanced CT	Contrast-enhanced CT and ultrasonography	Contrast-enhanced CT and ultrasonography	Contrast-enhanced CT
Site of thrombosis	Right IJV	Left IJV	Left IJV	Right IJV with extension into the sigmoid and transverse venous sinuses
Deep neck infection	Retropharyngeal abscess	Multiloculated neck abscess	Not documented	Carotid space abscess
Abscess drainage	Image-guided aspiration attempted (unsuccessful)	Not performed	Not applicable	Surgical incision and drainage
Microbiological findings	Blood cultures negative; no abscess culture	Blood cultures negative; no abscess culture	Blood cultures negative	Blood cultures negative; abscess culture positive for *Klebsiella pneumoniae*
Antibiotic therapy	Piperacillin/tazobactam followed by amoxicillin/clavulanate	Piperacillin/tazobactam followed by amoxicillin/clavulanate	Amoxicillin/clavulanate	Clindamycin and cefuroxime
Total antibiotic duration	4 weeks	Approximately 3 weeks	3 weeks	6 weeks
Anticoagulation	Apixaban 2.5 mg twice daily (planned for 3 months)	Prophylactic-dose enoxaparin	Apixaban 5 mg twice daily for 3 months	Warfarin for 6 weeks
Pulmonary septic emboli	None	None	None	None
Outcome	Resolution of deep neck infection at 2 weeks with persistent IJV thrombosis; complete thrombus resolution on subsequent follow-up imaging	Partial recanalization of the left IJV	Stable IJV thrombus without progression	Complete thrombus resolution at 6 weeks

ESRD, end-stage renal disease; CAD, coronary artery disease; CT, computed tomography; IJV, internal jugular vein.

## Discussion

Lemierre syndrome is characterized by septic thrombophlebitis of the IJV and may be complicated by metastatic septic emboli (10). Although it has historically been referred to as a “forgotten disease,” it continues to be reported worldwide. A prospective epidemiological study conducted in Denmark estimated an annual incidence of 3.6 cases per million population, while more recent studies suggest that the apparent incidence may be increasing due to improved diagnostic imaging and greater clinical recognition (5, 11, 12). Despite its rarity, Lemierre syndrome remains clinically important because of its potential for substantial morbidity and mortality.

Classically, Lemierre syndrome affects young, otherwise healthy adults. A large analysis of 712 patients reported a median age of 21 years (interquartile range, 17–33 years) (13). However, cases have also been described in pediatric and elderly populations (14, 15). In contrast to the classic demographic profile, patients in our series ranged from 31 to 72 years of age, with two patients older than 60 years and several having significant underlying comorbidities. These observations support the recognition that septic IJV thrombophlebitis can occur outside the traditional demographic profile and that advanced age or substantial comorbidity should not preclude consideration of Lemierre syndrome or related Lemierre-like presentations.

*Fusobacterium necrophorum* remains the pathogen most frequently associated with classic Lemierre syndrome, although other organisms, including *K. pneumoniae*, have been reported, particularly in patients with diabetes mellitus (4, 16). Microbiological confirmation was limited in our cohort: blood cultures were negative in all four patients, and only one abscess culture, obtained from Case 4, yielded *K. pneumoniae*. These findings should therefore not be interpreted as evidence of broad microbiological heterogeneity but rather as illustrating the diagnostic challenges encountered in culture-negative disease. Negative blood cultures may result from prior antibiotic exposure, delayed specimen collection, or difficulties in recovering anaerobic organisms, particularly *Fusobacterium* species (1, 6). Accordingly, negative blood cultures alone should not exclude the diagnosis when the clinical and radiological findings are otherwise compatible with septic IJV thrombophlebitis.

Classically, Lemierre syndrome develops following acute tonsillitis or peritonsillar infection, frequently caused by *F. necrophorum* (17). Other antecedent infections, including otitis media, mastoiditis, odontogenic infections, and deep neck space infections, have also been described (17). However, no universally accepted diagnostic criteria exist. Proposed definitions generally include a recent oropharyngeal infection, radiological evidence of IJV thrombosis, microbiological confirmation (typically *F. bacteremia*), and, frequently, metastatic septic emboli (1, 2). Contemporary literature also recognizes Lemierre-like presentations in which one or more of these classic features are absent (1, 6).

Our cases illustrate this diagnostic spectrum. Case 4 most closely represented classic Lemierre syndrome, with an antecedent oropharyngeal infection, a deep neck abscess, IJV thrombosis with intracranial extension, and microbiological confirmation from the abscess culture. Cases 1–3 were considered Lemierre-like presentations because they lacked one or more features typically associated with classic Lemierre syndrome. Case 1 developed septic IJV thrombophlebitis in close temporal association with Permcath insertion and in the absence of a preceding oropharyngeal infection, supporting a catheter-associated etiology. Case 2 presented with a deep neck abscess and IJV thrombosis without a documented antecedent oropharyngeal infection or microbiological confirmation. Case 3 developed IJV thrombophlebitis following an upper respiratory tract infection but had no documented deep neck abscess or microbiological confirmation. Thus, this series is best interpreted as representing a spectrum encompassing classic Lemierre syndrome and Lemierre-like septic IJV thrombophlebitis rather than exclusively classic disease.

Imaging plays a central role in the diagnosis and assessment of IJV thrombosis. Contrast-enhanced CT is commonly used as the first-line imaging modality because it can rapidly delineate the extent of thrombus, associated deep neck infection, and potential complications. MRI provides superior soft-tissue characterization and may be particularly useful when intracranial venous extension is suspected (1, 6). In our series, CT was the primary diagnostic modality in all patients, while ultrasonography contributed to diagnosis in selected cases and was also used for follow-up assessment. Follow-up imaging demonstrated a range of outcomes, including thrombus stability, partial recanalization, and complete resolution. Although there is no consensus regarding the optimal timing of surveillance imaging, repeat imaging may be useful in patients with persistent symptoms, extensive thrombosis, or those receiving anticoagulation to assess treatment response and identify thrombus progression (1, 6).

Antimicrobial therapy remains the cornerstone of treatment. Contemporary reviews generally recommend prolonged therapy, often for approximately 4–6 weeks, with the duration individualized according to disease severity and clinical response (1, 17). Empirical therapy should provide adequate anaerobic coverage, particularly against *F. necrophorum*. A *β*-lactam agent combined with metronidazole is commonly recommended because polymicrobial infection may occur, while metronidazole provides reliable anaerobic activity and tissue penetration (1, 17). Antimicrobial therapy may subsequently be tailored based on microbiological findings and clinical response, while maintaining appropriate coverage when cultures remain negative (1).

In our series, antimicrobial duration varied according to clinical presentation, disease extent, and treatment response. Case 4 received 6 weeks of antimicrobial therapy in the setting of extensive IJV thrombosis with intracranial venous sinus extension. Case 1 received 4 weeks of therapy, whereas Cases 2 and 3 received approximately 3 weeks. The shorter courses used for select patients reflected their clinical response, localized disease, and absence of documented metastatic septic complications rather than a standardized treatment protocol. These differences underscore the need to individualize antimicrobial duration according to the clinical course, particularly given the limited evidence defining optimal treatment duration.

The role of anticoagulation in Lemierre syndrome and Lemierre-like septic thrombophlebitis remains controversial because randomized controlled trials and evidence-based treatment guidelines are lacking (7, 8, 13). Anticoagulation is generally considered in selected patients, particularly in the presence of thrombus progression, persistent fever despite appropriate antimicrobial therapy, intracranial extension, embolic complications, or clinical deterioration (1, 7, 13). Surgical interventions, including IJV ligation or excision, have historically been described for refractory disease but have not demonstrated a consistent mortality benefit (12, 17).

Anticoagulation decisions in our series were individualized based on thrombus burden, anatomical extent, bleeding risk, comorbidities, and overall clinical status rather than following a uniform protocol. Case 4 received warfarin in the setting of extensive thrombosis extending into the sigmoid and transverse venous sinuses, where prevention of further thrombus propagation was considered clinically important. Case 3 received apixaban for IJV thrombosis in the absence of a documented contraindication to direct oral anticoagulant (DOAC) therapy. Case 2 received prophylactic-dose enoxaparin during hospitalization, as the thrombosis remained localized and clinical improvement occurred promptly with antimicrobial therapy. In Case 1, anticoagulation with apixaban was initiated because of septic IJV thrombosis in the setting of catheter-associated deep neck infection. These cases illustrate the individualized approach to anticoagulation, balancing the extent of thrombus and the risk of propagation against bleeding risk, comorbidities, and overall clinical status.

Although high-quality evidence remains limited, published reports, systematic reviews, and recent individual participant data analyses suggest that DOACs may represent a reasonable alternative to warfarin in selected patients with Lemierre syndrome who require anticoagulation (7, 8, 13). However, comparative evidence on anticoagulant selection remains limited, and there is no consensus on the optimal agent, treatment duration, or patient selection criteria. Potential benefits of anticoagulation include limiting thrombus propagation and promoting recanalization, but these benefits must be weighed against the risk of bleeding. Accordingly, anticoagulation decisions should remain individualized until higher-quality prospective evidence becomes available.

An important observation in our series is that none of the patients developed pulmonary septic emboli, a characteristic and clinically significant manifestation of classic Lemierre syndrome. The absence of pulmonary septic embolization distinguishes our cohort from many previously reported series and may reflect differences in disease extent, timing of diagnosis, or the inclusion of Lemierre-like presentations. Importantly, pulmonary septic emboli are not universally present, and their absence alone does not exclude Lemierre syndrome. Nevertheless, this feature, together with the absence of bacteremia in all four patients, warrants cautious interpretation of our findings and limits their generalizability to patients with disseminated classic Lemierre syndrome.

Overall, our case series illustrates the expanding clinical spectrum of septic IJV thrombophlebitis, encompassing both classic Lemierre syndrome and Lemierre-like presentations. The cases demonstrate that, particularly in culture-negative presentations, diagnosis may depend predominantly on the combination of compatible clinical features and characteristic radiological findings. They also highlight substantial variation in antimicrobial duration and anticoagulation strategies, reflecting the lack of standardized management approaches and the need to individualize treatment based on disease severity, thrombus extent, bleeding risk, comorbidities, and clinical response.

This study has several limitations. First, the small sample size and retrospective design limit the generalizability of the findings. Second, microbiological confirmation was limited: Blood cultures were negative in all four patients, and only one patient had a positive abscess culture. Third, none of the patients developed pulmonary septic emboli, limiting direct comparison with cohorts of disseminated classic Lemierre syndrome. Finally, three of the four cases were classified as Lemierre-like rather than as classic Lemierre syndrome because they lacked one or more of the classic clinical or microbiological features. These limitations highlight the diagnostic overlap between classic Lemierre syndrome and Lemierre-like septic IJV thrombophlebitis and underscore the need for standardized diagnostic criteria and higher-quality evidence to guide management.

## Conclusion

We presented a case series illustrating the changing clinical spectrum of septic internal jugular vein thrombophlebitis, ranging from classic Lemierre syndrome to Lemierre-like presentations. Our cases demonstrate that diagnosis can be made primarily on the basis of compatible clinical and radiological findings, despite negative blood cultures, underscoring the challenges of culture-negative disease. The absence of pulmonary septic emboli in this cohort further highlights the heterogeneity of contemporary Lemierre syndrome and Lemierre-like presentations. Management should be individualized based on disease severity, extent of thrombus, and patient-specific factors. Early initiation of appropriate antimicrobial therapy remains the cornerstone of treatment. Anticoagulation remains guided by clinical judgment, as there is insufficient high-quality evidence to support standardized indications, agent selection, or therapy duration. Larger prospective studies are needed to refine diagnostic criteria and optimize management strategies for classic Lemierre and Lemierre-like syndromes.

## Data Availability

The raw data supporting the conclusions of this article will be made available by the authors without undue reservation.
